# What Is the Role of [^18^F]FDG PET/CT in the “Recommendations for Updating Fever and Inflammation of Unknown Origin From a Modified Delphi Consensus Panel” by Wright et al?

**DOI:** 10.1093/ofid/ofae536

**Published:** 2024-09-14

**Authors:** Domenico Albano, Giorgio Treglia

**Affiliations:** Nuclear Medicine, ASST Spedali Civili Brescia, Brescia, Italy; Nuclear Medicine Department, University of Brescia, Brescia, Italy; Division of Nuclear Medicine, Imaging Institute of Southern Switzerland, Ente Ospedaliero Cantonale, Bellinzona, Switzerland; Faculty of Biomedical Sciences, Università della Svizzera italiana, Lugano, Switzerland; Faculty of Biology and Medicine, University of Lausanne, Lausanne, Switzerland

To the Editor—We read with great interest the recent publication by Wright et al [1] regarding the consensus-based recommendations for management of fever and inflammation of unknown origin (FUO/IUO).

We appreciate very much the efforts of the authors/expert panel and the design of this modified Delphi consensus, which involved several experts from different countries discussing crucial themes related to the definition, diagnosis, and treatment approaches of patients with FUO/IUO.

FUO/IUO is, by definition, a diagnostic challenge due to the fact that it is not a biologically uniform phenomenon but a manifestation of many disease processes of variable nature (infection, neoplasm, inflammatory, and/or miscellaneous). The idea to write an international consensus is brilliant and of clinical impact. One point discussed in this article is the potential yield of fluorine positron emission tomography/computed tomography ([^18^F]FDG PET/CT) in this setting. In our opinion, some discrepancies are present in the article about the role of [^18^F]FDG PET/CT. As a matter of fact, in the abstract, the authors wrote, “Experts strongly disagreed with using 2-deoxy-2-[18F] fluoro-D-glucose positron emission tomography/computed tomography as part of the diagnostic criteria for FUO.” This statement raises doubts about the usefulness of [^18^F]FDG PET/CT in the setting of FUO/IUO. However, this sentence seems to be in contradiction with the evidence-based results present in the literature and with the main text of the article by Wright et al [1]. For example, in Table 4, there is a clear consensus recommendation about the early use of [^18^F]FDG PET/CT in the management of FUO/IUO, especially if findings from plain radiographs or computed tomography are negative or nondiagnostic in the absence of potential diagnostic clues. Also, in the Discussion at “Theme 3: Evaluation—Consensus Recommendation 3,” the potential role of [^18^F]FDG PET/CT is well described, citing several meta-analyses that reported a significant diagnostic impact/clinical helpfulness in about 56% to 60% of cases [2–6], even better than conventional imaging [7].

These discordant findings in the abstract and full text are probably due to the difficulties in summarizing the main findings of this complex consensus. Additionally, in a recent consensus document of the European Association of Nuclear Medicine [8], the diagnostic role of [^18^F]FDG PET/CT was confirmed, taking into account available evidence-based data. Particularly, a positive [^18^F]FDG PET/CT scan was demonstrated to be useful for guiding treatment or subsequent diagnostic procedures, while a negative [^18^F]FDG PET/CT scan was usually associated with a spontaneous remission of disease and a favorable prognosis. Moreover, in specific populations, such as pediatrics, patients with HIV, and patients in intensive care units, the impact of [^18^F]FDG PET/CT seems to be high. The first author of this consensus [1] wrote an editorial to stress the usefulness of [^18^F]FDG PET/CT in FUO/IUO [9].

We believe that it is fundamental for the medical community to stress the evidence-based role of [^18^F]FDG PET/CT in FUO/IUO. Of course, more solid data are desirable to strengthen the evidence in favor of [^18^F]FDG PET/CT, but the evidence-based data available so far on this topic cannot be neglected [8] and are clearly superior when compared with expert opinions, even according to the pyramid of evidence [10].

## Notes

***Author contributions*.** All authors have seen and approved the letter.

## References

[1] Wright WF, Stelmash L, Betrains A, et al Recommendations for updating fever and inflammation of unknown origin from a modified Delphi consensus panel. Open Forum Infect Dis 2024; 11:ofae298. 10.1093/ofid/ofae298 PMC11222709 38966848

[2] Kan Y, Wang W, Liu J, Yang J, Wang Z. Contribution of 18F-FDG PET/CT in a case-mix of fever of unknown origin and inflammation of unknown origin: a meta-analysis. Acta Radiol 2019; 60:716–25. 10.1177/0284185118799512 30205705

[3] Takeuchi M, Dahabreh IJ, Nihashi T, Iwata M, Varghese GM, Terasawa T. Nuclear imaging for classic fever of unknown origin: meta-analysis. J Nucl Med 2016; 57:1913–9. 10.2967/jnumed.116.174391 27339873

[4] Besson FL, Chaumet-Riffaud P, Playe M, et al Contribution of (18)F-FDG PET in the diagnostic assessment of fever of unknown origin (FUO): a stratification-based meta-analysis. Eur J Nucl Med Mol Imaging 2016; 43:1887–95. 10.1007/s00259-016-3377-6 27037917

[5] Hao R, Yuan L, Kan Y, Li C, Yang J. Diagnostic performance of 18F-FDG PET/CT in patients with fever of unknown origin: a meta-analysis. Nucl Med Commun 2013; 34:682–8. 10.1097/MNM.0b013e328361cd0e 23636293

[6] Bharucha T, Rutherford A, Skeoch S, Alavi A, Brown M, Galloway J. Diagnostic yield of FDG-PET/CT in fever of unknown origin: a systematic review, meta-analysis, and Delphi exercise. Clin Radiol 2017; 72:764–71. 10.1016/j.crad.2017.04.014 28600002

[7] Ly KH, Costedoat-Chalumeau N, Liozon E, et al Diagnostic value of 18F-FDG PET/CT vs chest-abdomen-pelvis CT scan in management of patients with fever of unknown origin, inflammation of unknown origin or episodic fever of unknown origin: a comparative multicentre prospective study. J Clin Med 2022; 11:386. 10.3390/jcm11020386 PMC8779072 35054081

[8] Hess S, Noriega-Álvarez E, Leccisotti L, et al EANM consensus document on the use of [18F]FDG PET/CT in fever and inflammation of unknown origin. Eur J Nucl Med Mol Imaging 2024; 51:2597–613. 10.1007/s00259-024-06732-8 PMC11224117 38676736

[9] Wright WF, Kandiah S, Brady R, Shulkin BL, Palestro CJ, Jain SK. Nuclear medicine imaging tools in fever of unknown origin: time for a revisit and appropriate use criteria. Clin Infect Dis 2024; 78:1148–53. 10.1093/cid/ciae115 PMC11093677 38441140

[10] Sadeghi R, Treglia G. Systematic reviews and meta-analyses of diagnostic studies: a practical guideline. Clin Transl Imaging 2017; 5:83–7.

